# *Scutellaria barbata* D. Don Inhibits Tumor Angiogenesis via Suppression of Hedgehog Pathway in a Mouse Model of Colorectal Cancer

**DOI:** 10.3390/ijms13089419

**Published:** 2012-07-25

**Authors:** Lihui Wei, Jiumao Lin, Wei Xu, Qiaoyan Cai, Aling Shen, Zhenfeng Hong, Jun Peng

**Affiliations:** 1 Academy of Integrative Medicine Biomedical Research Center, Fujian University of Traditional Chinese Medicine, 1 Huatuo Road, Minhou Shangjie, Fuzhou, Fujian 350122, China; E-Mails: wlhlxing@hotmail.com (L.W.); linjiumao@gmail.com (J.L.); caiqiaoyan@fjtcm.edu.cn (Q.C.); shenaling@hotmail.com (A.S.); hong-zhenfeng@hotmail.com (Z.H.); 2 Fujian Key Laboratory of Integrative Medicine on Geriatrics, Fujian University of Traditional Chinese Medicine, 1 Huatuo Road, Minhou Shangjie, Fuzhou, Fujian 350122, China; 3 Department of Pharmacology, Fujian University of Traditional Chinese Medicine, 1 Huatuo Road, Minhou Shangjie, Fuzhou, Fujian 350122, China; E-Mail: xwfjtcm@sina.com

**Keywords:** *Scutellaria barbata* D. Don, Chinese medicine, colorectal cancer, angiogenesis, SHH pathway

## Abstract

Angiogenesis, which plays a critical role during tumor development, is tightly regulated by the Sonic Hedgehog (SHH) pathway, which has been known to malfunction in many types of cancer. Therefore, inhibition of angiogenesis via modulation of the SHH signaling pathway has become very attractive for cancer chemotherapy. *Scutellaria barbata* D. Don (SB) has long been used in China to treat various cancers including colorectal cancer (CRC). Our published data suggested that the ethanol extract of SB (EESB) is able to induce apoptosis of colon cancer cells and inhibit angiogenesis in a chick embryo chorioallantoic membrane model. To further elucidate the precise mechanisms of its anti-tumor activity, in the present study we used a CRC mouse xenograft model to evaluate the effect of EESB on tumor growth and angiogenesis *in vivo*. Our current data indicated that EESB reduces tumor size without affecting on the body weight gain in CRC mice. In addition, EESB treatment suppresses the expression of key mediators of the SHH pathway in tumor tissues, which in turn resulted in the inhibition of tumor angiogenesis. Furthermore, EESB treatment inhibits the expression of vascular endothelial growth factor A (VEGF-A), an important target gene of SHH signaling and functioning as one of the strongest stimulators of angiogenesis. Our findings suggest that inhibition of tumor angiogenesis via suppression of the SHH pathway might be one of the mechanisms by which *Scutellaria barbata* D. Don can be effective in the treatment of cancers.

## 1. Introduction

Formation of new blood vessels via angiogenesis is critical for the development of solid tumors [1,2]. Tumor angiogenesis is regulated by multiple cellular signaling pathways including Sonic Hedgehog (SHH) transduction cascade. SHH signaling is important for animal embryonic development [3] and its aberrant activation has been associated with many human cancers [4–6]. Activation of SHH signaling is initiated by binding of HH to the transmembrane receptor Patched-1 (PTCH-1). This results in the release of PTCH-mediated suppression of Smo, which consequently activates the Gli family of transcription factors that regulate the expression of HH target genes [7,8], including vascular endothelial growth factor A (VEGF-A) [9,10], which is considered to be the strongest stimulator of angiogenesis [11,12]. VEGF-A secreted from tumor cells primarily binds to specific receptors located on vascular endothelial cells (EC), such as VEGFR-2 [13,14], which in turn triggers a tyrosine kinase signaling cascade that induces EC proliferation, migration, sprouting and tube formation [11,15]. Therefore, inhibition of angiogenesis via modulation of SHH signaling could be a promising strategy for anti-cancer drug development.

Colorectal cancer (CRC) is one of the leading causes of death around the world. To date, chemotherapy is the main therapeutic approach for patients with advanced CRC; and 5-fluorouracil (5-FU)-based regimens continue to be the international standard chemotherapy for these patients. However, due to the drug resistance and the unacceptable level of toxicity to normal cells, systemic chemotherapy using 5-FU-based regimens produces objective response rates of less than 40% [16–18]. These problems highlight the urgent need for development of novel cancer chemotherapies. Recently, natural products, including traditional Chinese medicine (TCM), have received interest as they have relatively few side effects and have been used clinically for thousands of years as important alternative remedies for a variety of diseases [19,20]. *Scutellaria barbata* D. Don (SB) is a medicinal herb widely distributed in northeast Asia. As a well known traditional Chinese folk-medicine, it has long been used as an important component in several TCM formulas to treat various kinds of cancer [21–23]. It has been shown that extracts of SB (ESB) possess anti-tumor activity to suppress the growth of many types of cancer including CRC both *in vitro* and *in vivo* [24–30]. In addition, we previously reported that ESB promotes the apoptosis of human colorectal carcinoma HT-29 cells and inhibits angiogenesis *in vitro* [31,32]. To further elucidate the mechanism of the tumorcidal activity of *Scutellaria barbata* D. Don, here we investigated its *in vivo* anti-angiogenic activity as well as its effect on the SHH pathway.

## 2. Results and Discussion

### 2.1. EESB Inhibits Tumor Growth in CRC Xenograft Mice

**Figure 1:**
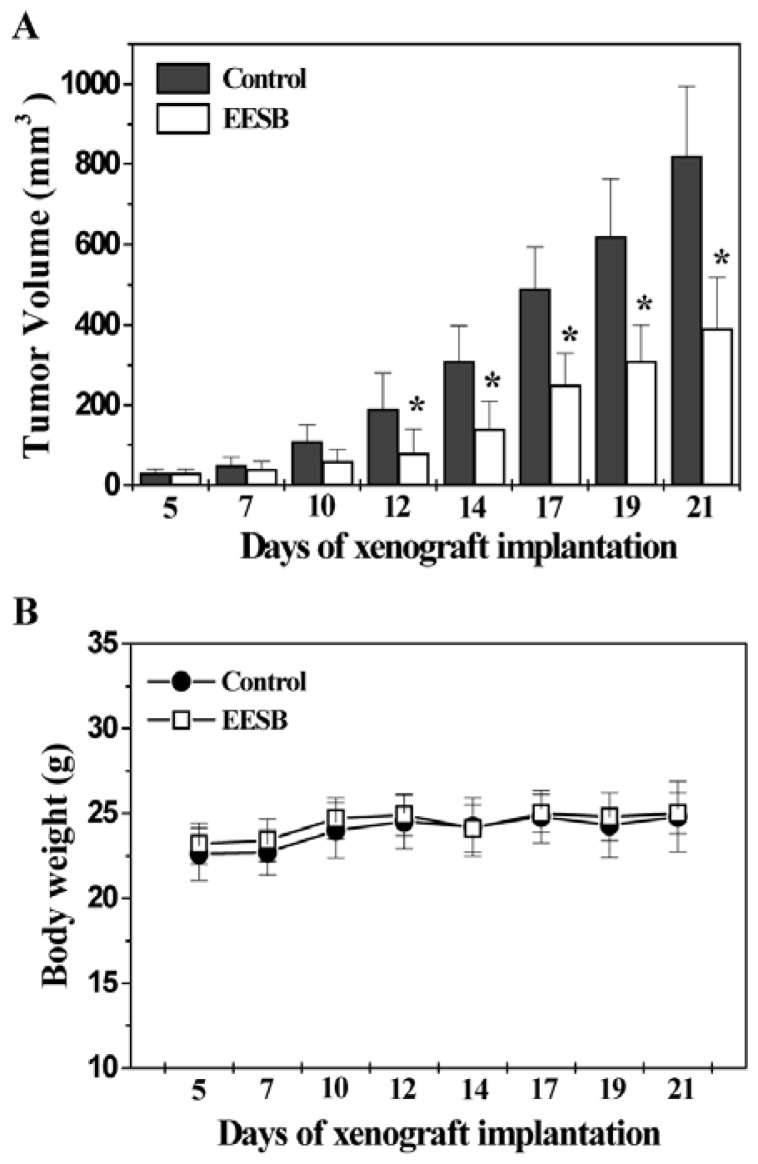
Effect of Ethanol extract of *Scutellaria barbata* D. Don (EESB) on tumor growth in colorectal cancer (CRC) xenograft mice. After tumor development, the mice were given intra-gastric administration with 2g/kg of EESB or saline daily, 5 days a week for 16 days. Tumor volume (**A**) and body weight (**B**) were measured during the experiment. Data shown were averages with S.D. (error bars) from 10 individual mice in each group. * *p* < 0.05, *versus* controls.

The *in vivo* efficacy of EESB against tumor growth was investigated by evaluating its effect on tumor volume in CRC xenograft mice, and its adverse effect was determined by measuring the body weight gain. As shown in Figure 1A, administration of EESB significantly decreased tumor weight in a time-dependent manner as compared with the control group (*p* < 0.05). In addition, EESB treatment did not affect animal body weight (Figure 1B). These data together suggest that EESB is potent in suppressing colon tumor growth *in vivo*, without apparent adverse effects.

### 2.2. EESB Inhibits Tumor Angiogenesis in CRC Xenograft Mice

**Figure 2:**
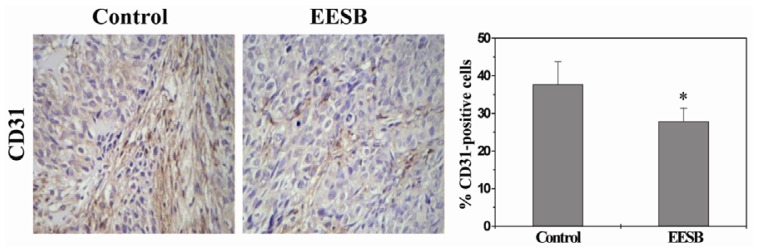
Effect of EESB on the intratumoral microvessel density in CRC xenograft mice. Tumor tissues were processed for immunohistochemical (IHC) staining for CD31. The photographs were representative images taken at a magnification of ×400. Quantification of IHC assay was represented as percentage of positively-stained cells. Data shown were averages with S.D. (error bars) from 10 individual mice in each group. * *p* < 0.05, *versus* controls.

Angiogenesis plays an important role in the development and metastasis of cancers. Our former published data indicated that EESB suppresses proliferation, migration and tube formation of endothelial cells and downregulates the expression of VEGF-A, which suggest that EESB may be involved in the regulation of angiogenesis *in vivo*. We therefore hypothesized that EESB inhibits tumor growth probably via suppressing angiogenesis. To test this hypothesis, we examined the effect of EESB on the intratumoral microvessel density (MVD) using immunohistochemical (IHC) staining for the endothelial cell-specific marker CD31. As shown in Figure 2, the percentage of CD31-positive cells in control and EESB-treated mice was 37.75 ± 5.95% and 27.83 ± 3.60%, respectively (*p* < 0.05), demonstrating that EESB-caused inhibition of colon tumor growth is accompanied by its anti-angiogenic activity.

### 2.3. EESB Suppresses SHH Signaling Pathway in CRC Xenograft Mice

**Figure 3:**
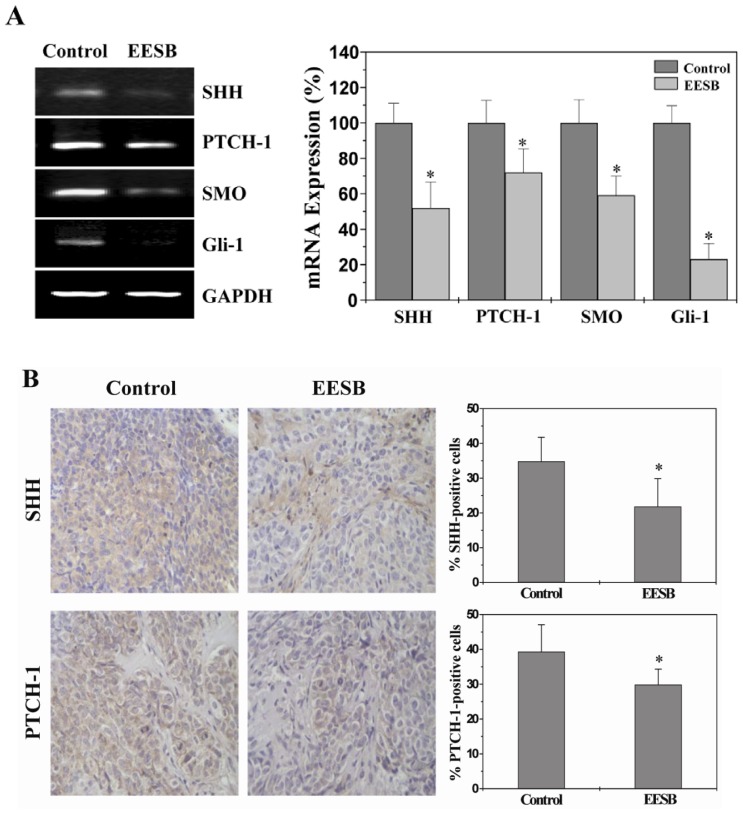

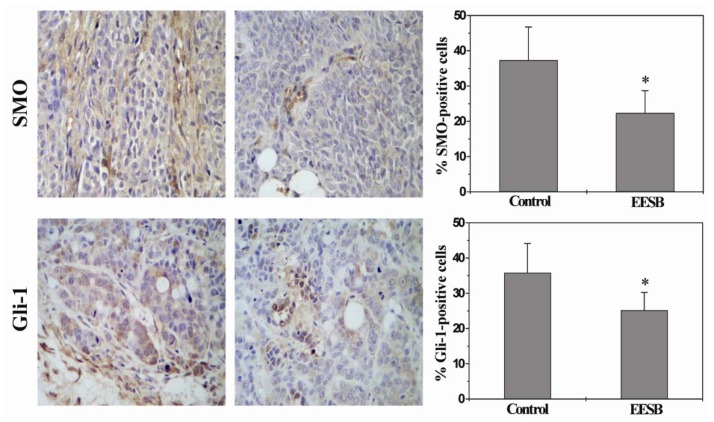
Effect of EESB on the activation of Sonic Hedgehog (SHH) pathway in CRC xenograft mice. (**A**) The mRNA expression levels of SHH, Patched-1 (PTCH-1, smoothened (SMO) and glioma-associated oncogene homolog 1 (Gli-1) were determined by RT-PCR. GAPDH was used as the internal control. The data of densitometric analysis were normalized to the mean mRNA expression of untreated control (100%); (**B**) Tumor tissues were processed for IHC staining for SHH, PTCH-1, SMO and Gli-1. The photographs were representative images taken at a magnification of ×400. Quantification of IHC assay was represented as percentage of positively-stained cells. Data shown were averages with S.D. (error bars) from 10 individual mice in each group. * *p* < 0.05, *versus* controls.

Tumor angiogenesis is tightly regulated by Sonic Hedgehog (SHH) pathway, we therefore assessed the effect of EESB on the expression of key mediators of SHH pathway using IHC and RT-PCR analyses. As shown in Figure 3A, EESB treatment profoundly reduced the mRNA expression of SHH, PTCH-1, SMO and Gli-1 in tumor tissues. Consistently, their protein expression was also significantly inhibited by EESB treatment. The percentage of SHH-, PTCH-1-, SMO- or Gli-1-positive cells in control group was 34.83 ± 6.91%, 39.33 ± 7.74%, 37.33 ± 9.40% or 35.83 ± 8.30%, whereas that in EESB-treated mice was 21.83 ± 8.04%, 29.83 ± 4.49%, 22.33 ± 6.31% or 25.17 ± 5.12% (Figure 3B). Collectively, these data suggest that the *in vivo* inhibitory effect of EESB on tumor angiogenesis could be mediated by the suppression of SHH signaling pathway.

Like most of other medicinal herbs, *Scutellaria barbata* D. Don (SB) is composed of many chemical components such as flavonoids, diterpenoids, alkaloids, steroides and polysaccharides. Herbal medicines including SB are thus considered to be multi-target agents that exert their therapeutic function in a more holistic way, which might be one of the reasons that EESB inhibits HH pathway in multiple levels.

### 2.4. EESB Inhibits the Expression of VEGF-A and VEGFR2 in CRC Xenograft Mice

**Figure 4:**
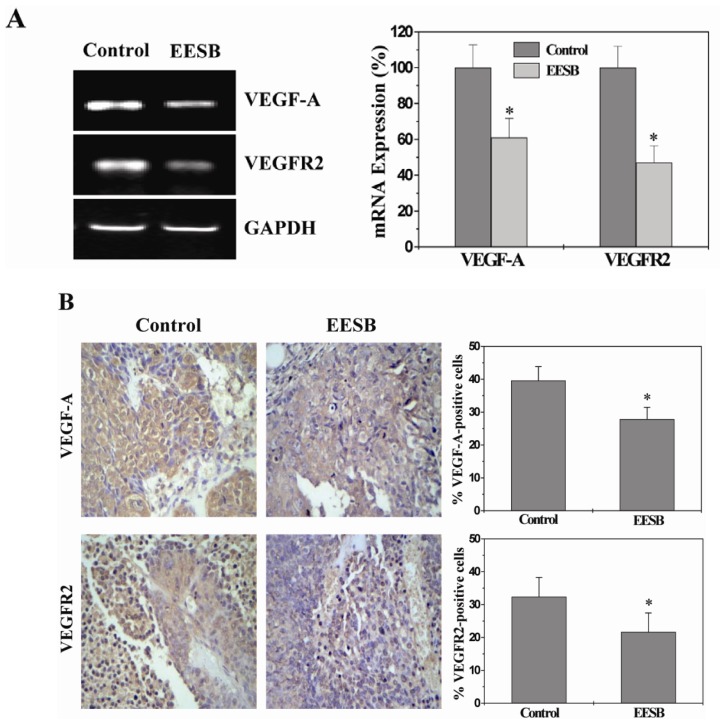
Effect of EESB on the expression of VEGF-A and VEGFR2 in CRC xenograft mice. (**A**) The mRNA expression levels of Vascular endothelial growth factor-A (VEGF-A) and VEGF Receptor 2 (VEGFR2) were determined by RT-PCR. GAPDH was used as the internal control. The data of densitometric analysis were normalized to the mean mRNA expression of untreated control (100%); (**B**) Tumor tissues were processed for IHC staining for VEGF-A and VEGFR2. The photographs were representative images taken at a magnification of ×400. Quantification of IHC assay was represented as percentage of positively-stained cells. Data shown were averages with S.D. (error bars) from 10 individual mice in each group. * p < 0.05, *versus* controls.

Activation of HH signaling up-regulates the expression of VEGF-A which is one of the strongest angiogenesis stimulators [15–19]. VEGF-A exerts its pro-angiogenic function via binding to its specific receptors including VEGFR2 which is located on vascular endothelial cells (EC) [17,20,23], leading to series of angiogenic processes [17,24]. To further explore the mechanism of EESB’s anti-angiogenic activity, we performed RT-PCR and IHC analyses to respectively examine the mRNA and protein expression of VEGF-A and VEGFR2 in CRC mice. Results of the RT-PCR showed that EESB treatment reduced the mRNA expression of VEGF-A and VEGFR2 in tumors (Figure 4A). Data from IHC assay indicated that the protein expression patterns of VEGF-A and VEGFR2 were similar to their respective mRNA levels. The percentage of VEGF-A-and VEGFR2-positive cells in control group was 39.60 ± 4.22% and 32.40 ± 5.87%, while that in EESB-treated mice was 27.83 ± 3.60% and 21.67 ± 5.78% (Figure 4B).

## 3. Experimental Section

### 3.1. Materials and Reagents

Dulbecco’s Modified Eagle Medium (DMEM), Fetal bovine serum (FBS), penicillin–streptomycin, trypsin-EDTA, and TriZol reagent were purchased from Invitrogen (Carlsbad, CA, USA). SuperScript II reverse transcriptase was obtained from Promega (Madison, WI, USA). CD31, SHH, PTCH-1, SMO, Gli-1, VEGF-A and VEGFR2 antibodies, secondary antibodies were obtained from Cell Signaling (Beverly, MA, USA). All other chemicals, unless otherwise stated, were obtained from Sigma Chemicals (St. Louis, MO, USA).

### 3.2. Preparation of Ethanol Extract from *Scutellaria barbata* D. Don

Authentic plant material was purchased from Guo Yi Tang Chinese Herbal medicine store, Fujian, China. The original herb was identified as *Scutellaria barbata* D. Don (SB) by Wei Xu at Department of Pharmacology, Fujian University of Traditional Chinese Medicine, China. Ethanol extract of SB (EESB) was prepared as previously described [31]. Briefly, 1 kg of SB were extracted with 8 L of 85% ethanol using refluxing method and filtered. The ethanol solvent was then evaporated on a rotary evaporator (Yarong, Model RE-2000, Shanghai, China). The resultant solution was concentrated to a relative density of 1.10, and the dried powder of EESB was obtained by spraying desiccation method using a spray dryer (Buchi, Model B-290, Switzerland). The working concentrations of EESB were made by dissolving the extractum in saline to a concentration of 0.5 g/mL.

### 3.3. HPLC Analysis

**Figure 5:**
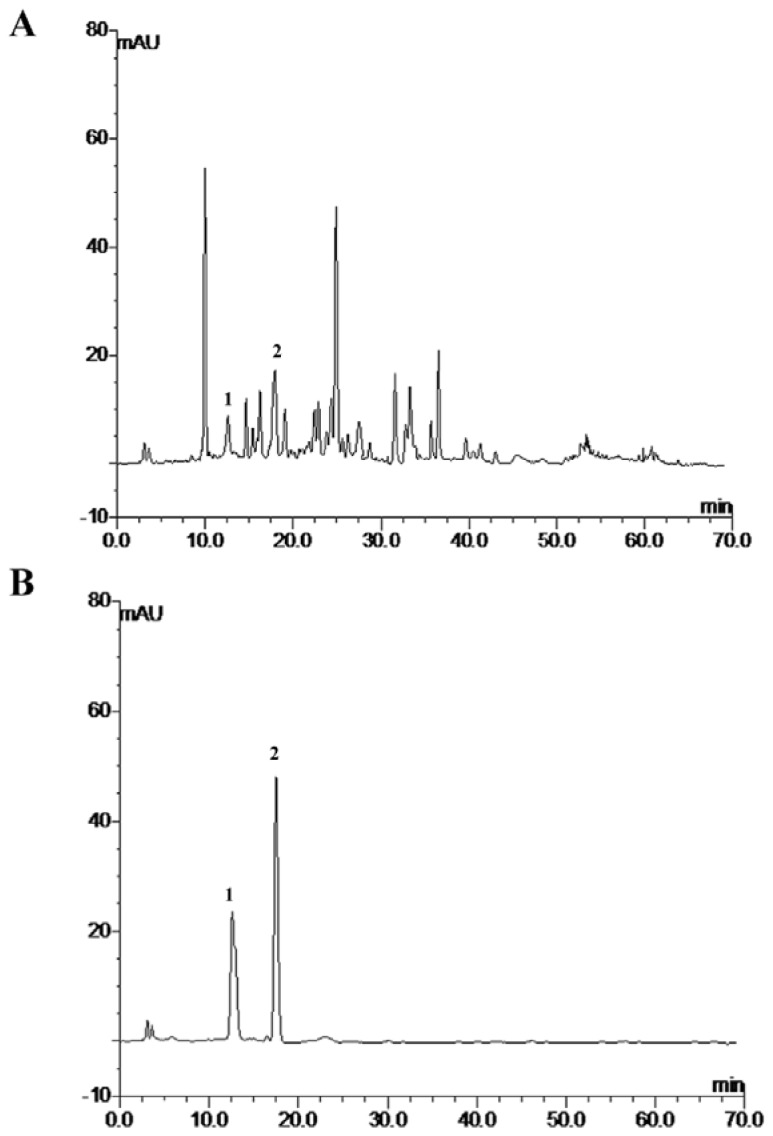
HPLC profiles of EESB (**A**) and a control sample (**B**). The mobile phase consisted of methanol:water:acetic acid = 35:61:4. The control sample was composed of scutellarein (peak 1) and baicalin (peak 2).

EESB was analyzed on an Agilent 1100 HPLC system (Agilent, Santa Clara, CA, USA) using a C-18 column (4.6 mm × 150 mm, 5 μm). Absorbance was measured at 355 nm (Figure 5). The mobile phase consisted of methanol: water: acetic acid at 35:61:4 at a flow rate of 1 mL/min with an injection volume of 10 μL. A sample containing scutellarein and baicalin was used as a control.

### 3.4. Cell Culture

Human Colon carcinoma cell line HT-29 was obtained from the American Type Culture Collection (ATCC, Manassas, VA, USA) and grown in DMEM medium supplemented with 10% (v/v) FBS, 100 units/mL penicillin and 100 μg/mL streptomycin in a 37 °C humidified incubator with 5% CO_2_.

### 3.5. Animals

Athymic BALB/c nu/nu male mice (with an initial body weight of 20–22 g) were obtained from Shanghai SLAC Laboratory Animal Co., Ltd. (Shanghai, China) and housed under pathogen-free conditions with a 12 h light/dark cycle. Food and water were given *ad libitum* throughout the experiment. All animal treatments were strictly in accordance with international ethical guidelines and the National Institutes of Health Guide concerning the Care and Use of Laboratory Animals, and the experiments were approved by the Institutional Animal Care and Use Committee of Fujian University of Traditional Chinese Medicine.

### 3.6. *In vivo* Nude Mouse Xenograft Formation

HT-29 cells were grown in culture and then detached by trypsinization, washed, and resuspended in serum-free DMEM. 1.5 × 10^6^ of cells mixed with Matrigel (1:1) were subcutaneously injected in the right flank area of athymic nude mice to initiate tumor growth. After 5 days of xenograft implantation, mice were randomized into two groups (*n* = 10) and given intra-gastric administration with 2 g/kg of EESB or saline daily, 5 days a week for 16 days. Body weight and tumor growth were measured every two days. Tumor growth was determined by measuring the major (L) and minor (W) diameter with a caliper. The tumor volume was calculated according to the following formula: tumor volume = p/6 × L × W^2^. At the end of experiment, the animals were anaesthetized and the tumor tissue was removed.

### 3.7. Immunohistochemistry Analysis

After fixed with 10% formaldehyde for 12 h, tumor samples were processed conventionally for paraffin-embedded tumor slides. The slides were subjected to antigen retrieval and the endogenous peroxidase activity was quenched with hydrogen peroxide. After blocking non-specific proteins with normal serum in PBS (0.1% Tween 20), slides were incubated with rabbit polyclonal antibodies against CD31, SHH, PTCH-1, SMO, Gli-1, VEGF-A and VEGFR2 (all in 1:100 dilution, Santa Cruz Biotechnology). After washing with PBS, slides were incubated with biotinylated secondary antibody followed by conjugated horseradish peroxidase (HRP)-labelled streptavidin (Dako), and then washed with PBS. The slides were then incubated with diamino-benzidine (DAB, Sigma) as the chromogen, followed by counterstaining with diluted Harris hematoxylin (Sigma). After staining, five high-power fields (400×) were randomly selected in each slide, and the average proportion of positive cells in each field were counted using the true color multi-functional cell image analysis management system (Image-Pro Plus, Media Cybernetics, Bethesda, MD, USA). To rule out any nonspecific staining, PBS was used to replace the primary antibody as a negative control.

### 3.8. RNA Extraction and RT-PCR Analysis

Total RNA was isolated from fresh tumor with TriZol Reagent. Oligo(dT)-primed RNA (1 μg) was reverse-transcribed with SuperScript II reverse transcriptase according to the manufacturer’s instructions. The obtained cDNA was used to determine the mRNA amount of SHH, PTCH-1, SMO, Gli-1, VEGF-A, VEGFR2 by PCR. GAPDH was used as an internal control. The sequences of the primers used for amplification of SHH, PTCH-1, SMO, Gli-1, VEGF-A, VEGFR-2 and GAPDH transcripts are as follows: SHH forward 5′-CGC AGC GAG GAA GGG AAA G -3′ and reverse 5′-TTG GGG ATA AAC TGC TTG TAG GC -3′; PTCH-1 forward 5′-CGG CGT TCT CAA TGG GCT GGT TTT -3′ and reverse 5′-GTG GGG CTG CTG TTT CGG GTT CG -3′; SMO forward 5′-ATC TCC ACA GGA GAG ACT GGT TCG -3′ and reverse 5′-AAA GTG GGC CTT GGG AAC ATG -3′; Gli-1 forward 5′-TCT GCC CCC ATT GCC CAC TTG -3′ and reverse 5′-TAC ATA GCC CCC AGC CCA TAC CTC- 3′; VEGF-A forward 5′-CAT CCT GGC CTC GCT GTC -3′ and reverse 5′-CTC GCT CCA ACC GAC TGC -3′; VEGFR-2 forward 5′-TGG CTC ACA GGC AAC ATC -3′ and reverse 5′-CTT CCT TCC TCA CCC TTC -3′; GAPDH forward 5′-CG ACC ACT TTG TCA AGC TCA -3′ and reverse 5′-AG GGG TCT ACA TGG CAA CTG -3′.

### 3.9. Statistical Analysis

Data were presented as mean ± SD for the indicated number of independently performed experiments. The statistical analysis was carried out using the Student’s t test and *p* < 0.05 was considered to be statistically significant.

## 4. Conclusions

In conclusion, here for the first time we demonstrate that *Scutellaria barbata* D. Don inhibits colorectal cancer growth *in vivo* via inhibition of SHH-mediated tumor angiogenesis, which may in part explain its anti-cancer activity.
